# Water Drinking Behavior Associated with Aversive Arousal in Rats: An Integrative Approach

**DOI:** 10.3390/brainsci13010060

**Published:** 2022-12-29

**Authors:** Stefan M. Brudzynski

**Affiliations:** Department of Psychology, Brock University, St. Catharines, ON L2S 3A1, Canada; sbrudzynski@brocku.ca

**Keywords:** carbachol, atropine, intracerebral drug application, polydipsia, cholinergic system, ascending mesolimbic cholinergic system, aversive arousal, rat

## Abstract

Cholinergic muscarinic stimulation of vast areas of the limbic brain induced a well-documented polydipsia in laboratory rats. This excessive water-drinking behavior has not received any convincing biological and physiological interpretation for the last 50 years. This review offers such an interpretation and suggests that cholinergically induced drinking response, mostly by carbachol, is associated with activation of the ascending mesolimbic cholinergic system that serves for initiation of emotional aversive arousal of the organism. The ascending cholinergic system originates from the laterodorsal tegmental nucleus, has a diffuse nature, and affects numerous subcortical limbic structures. It is proposed that the carbachol-induced drinking response is related to the state of anxiety and does not serve the regulation of thirst. Instead, the response is anxiety-induced polydipsia that might occur as a soothing procedure that decreases the aversiveness of the negative emotional state induced by carbachol. It is concluded that carbachol-induced water-drinking behavior is a rewarding process that contributes to alleviating the feeling of anxiety by bringing some relief from the cholinergically induced aversive state, and it is a homologue to anxiety-driven polydipsia in humans.

## 1. History Background

Direct cholinergic activation of numerous areas of the forebrain and diencephalic limbic structures consistently induced polydipsia, i.e., significant water ingestion in rats that have previously drunk to satiation [1,2,3,4,5,6,7,8,9]. The drinking response was induced by muscarinic compounds such as carbachol, pilocarpine, muscarine, or physostigmine, was dose-dependent, and reversed by intracerebral atropine or systemic application of centrally active atropine salt, as well as, by scopolamine or methylatropine, but not by hexamethonium or d-tubocurarine [8,10,11,12,13,14,15,16]. Any method of drug delivery, e.g., in crystalline form, by solution injection, or by iontophoretic application, caused comparable responses [1,2,4,5,16,17]. For intracerebral injections, the drinking response was also not affected by the solvent used (water, saline, or sucrose solutions) [15].

In the studies of the cholinergic drinking response, carbachol was used most frequently because of its stability and long-lasting effects. The carbachol-induced drinking response in rats was specific to this neurochemical activation [18], and drinking was not successfully induced by electrical stimulation of the brain sites, from which carbachol-induced drinking. Carbachol-induced drinking response was consistently reproduced for the rat brain; however, it was not obtained from the brains of monkeys [19,20], cat [21,22], guinea pig [9], chicken, or dove [23,24], while a marginal and unreliable drinking response was reported for dogs after intracerebroventricular injection of carbachol [25]. In the rabbit, a limited increase in drinking after carbachol was obtained from the supraoptic nucleus only [26], and in the sheep brain, drinking was obtained with only 14% efficacy [27]. Intracerebroventricular injection of carbachol in newborn rat pups had no effect on drinking before the 4th postnatal day [28]. The carbachol-induced drinking was not a “blind”, reflexive drinking, but it was augmented when a palatable saline solution was presented and decreased with unpalatable concentrated solutions [17]; and the response could be conditioned [29].

This robust pharmacological muscarinic response was characterized by two interesting features. It could be antagonized by muscarinic antagonist, atropine, not only at the carbachol injection site, but it could be also antagonized from a different ipsilateral site or even from contralateral structures to the injection site [30,31,32]. It was also noticed that atropine mostly blocked carbachol effects when it was applied caudally to the carbachol site and only weakly worked when it was applied rostrally to the carbachol site. The second feature was that the drinking response could be induced from vast areas of the basal forebrain and diencephalon such as lateral septum, diagonal band, preoptic area, anterior hypothalamic area, other hypothalamic nuclei, subfornical organ, hippocampus, region of the fornix-hippocampal commissure and corpus callosum, vertical diagonal band, ventral amygdala, cingulate cortex, as well as, anterior, anteroventral and medial thalamus, medial midbrain, and also from cerebral ventricles [5,10,13,32,33,34,35,36].

The biological and physiological roles of this well-documented pharmacological response have not been offered and convincingly interpreted for the last 50 years because of serious problems with explaining many confusing observations. This strong and persistent behavioral response must be originating from a muscarinic activation of an important and widespread brain system. The present review will suggest a hypothesis, which will attempt to elucidate all the puzzling results, which are described below, and will suggest a possible explanation of this phenomenon.

## 2. The Problems

The finding of the cholinergically induced drinking response posed a problem in explaining specific role of the cholinergic connections in the extensive local limbic circuitries, and particularly explaining its role in physiological regulation of thirst. It was initially speculated that carbachol injected into the brain diffuses to cerebral ventricles and works from there on numerous structures, hence intracerebroventricular application of this drug increased drinking response [37,38]. This “ventricular hypothesis”, however, was not supported by further research and was disproved [34,39,40,41]. It was even suspected that the effects of intracerebral carbachol on drinking might be “*some sort of artifact and not really involved in normal hunger or thirst*” [42] (p. 11) because the effects of carbachol were lacking anatomical specificity.

Further experiments, comparing the effects of salt-induced drinking and carbachol-induced drinking led to the conclusion that carbachol is not mimicking the natural brain mechanisms of drinking induced by salt injection or water deprivation [31]. Intracranial injection or infusion of carbachol caused a transient increase in water intake but had no effect on the intake of NaCl [43]. Thus, the drinking induced by carbachol was not comparable with mechanisms of drinking resulting from water deprivation, cellular dehydration, or hypovolemia and seemed not to be relevant to homeostatic water balance mechanisms [9]. It was also suggested that the cholinergic drinking phenomenon has not only little relationship to natural thirst but may be also not relevant to species other than rat [9,44].

A peptide hormone angiotensin II, a part of the renin-angiotensin system [45,46] for regulation of body fluids and electrolytes, which is also a dipsogen and can induce drinking response in rats, worked at a lower threshold dose than that for carbachol, lower maximum effective dose, and with shorter latency [15]. Although atropine abolished carbachol-induced drinking, it had no effect on angiotensin II-induced drinking [36,47]. It was also shown that angiotensin and carbachol work on independent receptors in producing the drinking response [48], and that the renin-angiotensin system is not involved in the cardiovascular effects and drinking response induced by cholinergic stimulation [49]. Thus, the question remained why rats drank water after intracerebral application of carbachol?

It was expected that electrolytic lesions of the brain sites, from which carbachol induced drinking, could help in solving the problem. Effects of electrolytic lesions placed in the lateral septum, from which carbachol induced strong drinking response (positive site), were also not entirely clear. Unilateral, but also bilateral lesions had similar decreasing effect on drinking. Moreover, lesioning of a negative carbachol drinking site was as effective in decreasing drinking as lesion of a positive site [50]. Decrease in drinking was, however, related to the size of the lesion but was not related to the location of the lesion as studied in the region of the lateral septum, and drinking could be still elicited from about 50% of the lesioned sites [50]. Atropine applied to the lesioned site could block carbachol-induced drinking, even though lesioning was not, and could block the drinking response from a non-lesioned site as well. Therefore, it was concluded that atropine did not directly block cholinergically indued drinking by acting on the local neural activity [50]. Additionally, lesions of many other brain structures that induced drinking after cholinergic stimulation, did not disrupt the normal pattern of thirst behavior [51].

## 3. Specific Activation of a Diffuse Cholinergic System

Cholinergic stimulation induced drinking response and significant water intake from many interrelated limbic and diencephalic structures in the rat. This observation led to the initial suggestion that a generalized Papez circuit mediates the response, and this circuit is specifically sensitive to cholinergic activation [3].

The fact that the low threshold drinking sites were widely distributed throughout the subcortical limbic regions, and that the intracerebral atropine blocked the carbachol drinking response not only from the injection site but also from other distant limbic sites suggested activation of a widespread and interconnected, diffuse cholinergic system [9,12]. Electrophysiological multiunit studies confirmed that carbachol-induced drinking seems to result from the activity of a diffuse cholinergically coded circuit [52].

Extensive, diffuse, ascending cholinergic fibres originating from the brain stem have been described in numerous studies. Cholinergic innervation of the diencephalic and basal forebrain regions originates from tegmentum, mostly from the laterodorsal tegmental nucleus (LDT) as studied in rats and cats [53,54,55,56]. Further studies with immunochemical labeling for the vesicular acetylcholine transporter protein, a specific marker for cholinergic synapses [57,58], provided more detailed and specific data on these diffuse ascending projections to the basal forebrain, diagonal band, septum, hypothalamus, and other structures [59]. Initial anatomical studies of lateral septum, which was directly involved in inducing drinking response by carbachol, postulated direct cholinergic innervation of the lateral septum by neurons of the LDT [60]. In later studies, using a fluorescent retrograde tracer, fluorogold, and immunostaining for choline acetyltransferase (ChAT), a direct connection from the cholinergic neurons in the LDT to the lateral septum was confirmed [61].

The anterograde and retrograde anatomical tracing techniques combined with ChAT staining provided evidence that the LDT (and some adjoining cholinergic neurons to the LDT) supply substantial portion of the ascending mesolimbic tegmental afferents to the hypothalamus and lateral septum, and other structures, from which drinking response was obtained. Moreover, the ascending cholinergic connections were shown to release acetylcholine in the forebrain areas in freely moving rats [62]. These cholinergic fibres have multiple varicosities suggesting numerous synaptic contacts and large terminal fields originating from a single neuron in the LDT [60]. Similar cholinergic diffuse connections from the LDT to thalamus, including the reticular thalamic nuclei, and to the subthalamic nucleus were demonstrated [63,64]. As revealed by ultrastructural studies of the ascending cholinergic fibres to the anteroventral thalamic nucleus, the fibres had small terminals with symmetrical and asymmetrical contacts on different postsynaptic cellular components [65]. Some heterogeneity of the cholinergic innervation was noted in an immunocytochemical study both within and among different brain regions, which is characteristic for a diffuse pattern of cholinergic innervation [66].

The ascending cholinergic fibres have interesting features. They were reported frequently branching, so the unilaterally injected tracer into the LDT showed bilateral connections with a vast area of the midbrain and the forebrain, including the pontine reticular formation and the periaqueductal gray [53,67,68,69]. It was found that an average of 8% of the total population of cholinergic neurons in the LDT and the neighbouring pedunculopontine nucleus have, at least, bifurcating axons and dual projections to the thalamus and basal forebrain regions.

The ascending cholinergic projections from the LDT and neighbouring areas radiate to the forebrain [70] and are subjected to a large degree of collateralization. The thickest axons are gradually thinner on their way, suggesting numerous axon collaterals [55]. Many small collaterals arise from the primary axon at right angles and by further branching create terminal plexuses with extremely fine fibers with numerous varicosities as it was observed right in the LDT in brain slice preparations [71]. Such dense cholinergic plexuses with synapsing varicosities were confirmed in terminal fields of other structures innervated by cholinergic fibers and were described in detail in the lateral geniculate complex of macaque monkey [72].

This pattern of diffuse ascending cholinergic connections explains why stimulation of so many basal forebrain and diencephalic sites generated similar behavioral response, i.e., drinking. In other words, all the diffusely distributed cholinergic terminals have common behavioral function. Thus, activation of a sufficient portion of that ascending system from any location innervated by this system brings about similar response. However, the anatomical pattern of these projections is not explaining why the drinking response initiated from one site of the brain could be antagonized by atropine from a different, distant location other than the injection site.

## 4. Cholinergic Component of the Ascending Reticular Activating System

The ascending cholinergic connections from the LDT form part of the ascending pathways of the reticular activating system [73,74,75,76,77]. The LDT houses a concentration of cholinergic neurons labeled as Ch6 with long ascending projections. Some neurons belonging to the neighbouring Ch5 neuronal groups in the brainstem have also long ascending projections [78]. These cholinergic neurons have certain common morphological features as perikaryal heteromorphism and isodendritic arborization [79]. The isodendritic arborization is a feature of pluripotent neurons with overlapping dendritic fields in the reticular core. These neurons can process afferent signals of heterogenous origin [80] and are a feature of reticular formation neurons in the entire reticular core of the brainstem [81].

Although, the groups of brainstem cholinergic neurons were initially not included in the ascending reticular activating system, it appeared that these cells, together with other GABA-ergic and glutamatergic neurons form a functional pontomesencephalic cholinergic unit that is involved in arousal and regulation of numerous functions [82,83]. The detailed understanding of functioning of the arousal system was complicated by lack of clarity as to what roles should be ascribed to numerous groups of neurons within the ascending pathways (including pontomesencephalic nuclei) that, in addition to the cholinergic system, synthesize different transmitters and release them in vast areas of the brain and in the entire neocortex (for review see, [84,85]).

The cholinergic neurons of the LDT, however, provide a large proportion of mesopontine tegmental afferents to many limbic subcortical structures of the brain [60], and these afferents are regarded as a substantial component of the brain arousal system and component of the ascending reticular activating system [75,84,86]. The ascending cholinergic projections are mainly targeted at the brainstem limbic and basal forebrain limbic structures and relay stations (e.g., habenula; [87]). These projections contribute to the arousal with a limited cortical input, which reaches only the infralimbic and cingulate cortices as studied in rats and mice [54,83]. It has been found using the localized lesion technique that the classical reticulo–thalamo–cortical pathway for general cortical arousal may play a limited role in behavioral or electrocortical arousal in the rat brain, while projections from the pontomesencephalic tegmental nuclei, such as parabrachial nucleus and neighboring region including LDT, were more critical for arousal [88].

## 5. The Ascending Mesolimbic Cholinergic System for Aversive Arousal

The existence of two functionally discernable arousal system has been suggested long time ago [89]. One of these systems (named Arousal System I) was cortically oriented and associated with arousal in response to all environmental stimuli, while the other (Arousal System II) was related to the limbic system and associated with incentive-related stimuli [89]. The operation of the limbic system as an arousal system was, however, unclear. It was further complicated by very elaborate models of its arousing functions [90]. Moreover, neurochemical, and behavioral studies suggested inclusion of many ascending transmitter systems (noradrenalin, acetylcholine, dopamine, and serotonin) in the regulation of arousal, and the cholinergic component was postulated to be responsible for stimulus detection [91]. Further studies provided descriptions of numerous ascending projections based on many neurotransmitters, such as glutamatergic, cholinergic, noradrenergic, dopaminergic, serotonergic, histaminergic, and orexinergic systems, which influence the activity of the neocortex, basal forebrain, and activity of the limbic system [85]. This multitude of extensive ascending projections led to the conclusion of the existence of multiple arousal systems, which are interrelated and have combined arousing action on the brain [92,93,94]. The projections of the cholinergic component of the ascending afferents, particularly to the limbic structures were neglected and interpreted in terms of only cortical arousal [95].

All these hypotheses were not clearly distinguishing between cognitive arousal and emotional arousal, although, the emotional component of arousal associated with the effects of intracerebral carbachol was suggested some time ago by Grossman [96], who first reported carbachol-induced drinking response. He stated that “*it seems that the diencephalon may contain a very diffuse system of cholinergic fibers and cells, which seem to be part of the neural mechanisms which control affective reactions*” [96] (p. 79).

Many decades of thorough studies with pharmacological approach to emission of rat ultrasonic vocalization provided a new model that explained affective arousal and its relationship to the aversive or appetitive nature of these responses. Vocalization expresses animal emotionality [97,98,99] and is particularly suitable for studying emotional behavior and for developing new treatments for affective disorders [100]. Extensive functional mapping and anatomical tracing studies (e.g., [61,101,102]), revealed a two-component emotional arousal system that was based on the ascending cholinergic projections for aversive arousal and on the ascending dopaminergic projections for appetitive arousal [103,104]. Thus, the diffuse ascending cholinergic projections are associated with emotional aversive arousal [105], and these projections are of special importance for this review.

This ascending acetylcholine system originates from cholinergic perikarya located mostly in the medial part of LDT [82] and has been termed ascending mesolimbic cholinergic system for aversive emotional arousal [105,106]. It innervates predominantly the medial diencephalic and basomedial forebrain regions [53,54,56] and is reaching to the medial prefrontal cortex [107]. In addition to that, it has been shown using c-Fos labeling and immunohistochemical staining of cholinergic cell bodies for ChAT, that the cholinergic neurons of the LDT are active during initiation of aversive responses with emission of alarming 22 kHz vocalizations in rats [108]. Functional mapping of vocal responses that were induced by intracerebral injections of carbachol in the rat and cat brains revealed an extensive subcortical system of cholinoceptive limbic regions [101,109] that were comparable to the extensive regions, from which the drinking response was obtained in the rat. 

Thus, it may be postulated that the drinking response (polydipsia) induced in rats by carbachol was caused by activation of the emotional aversive arousal system, which initiated an emotional state, and was not associated with the regulation of thirst. This hypothesis would explain most problems the researchers had when they tried explaining the carbachol-induced drinking response as a homeostatic regulation of thirst. 

The ascending mesolimbic cholinergic system for aversive emotional arousal has diffused nature and single LDT neuron innervates many structures on the ipsilateral and contralateral side of the brain [53,56,69,110]. It was hypothesized long time ago that the forebrain limbic regions form reciprocal loops with the midbrain limbic regions [111], so the forebrain limbic structures with terminal fields of the ascending cholinergic projections and the LDT are reciprocally interconnected. Hence, activation of the terminal fields by carbachol would reciprocally activate the source neurons of the LDT [108,111]. This was directly proven by studies using c-Fos for labeling active neurons and ChAT immunohistochemical labeling of cholinergic neurons in the LDT. Injection of carbachol into forebrain sites activated cholinergic neurons within the LDT, i.e., the source neurons of the ascending projections [108]. This important finding may explain why injection of atropine, a cholinergic muscarinic antagonist, into the brain sites different than the original site of carbachol-activated drinking response (including contralateral sites) were able, at least partially, to antagonize the response, presumably by blocking the reciprocal connections.

## 6. The Set of Symptoms of Aversive Arousal and Their Biological Role

Experiments with direct cholinergic stimulation of the ascending cholinergic projections have revealed a vast array of defensive responses comprising both somatic and autonomic symptoms. They include general arousal, preparation of the motor and sensory systems for immediate action such as fight or escape, activation of the autonomic and endocrine systems, and emergency activation of bodily resources. Intracerebral injection of carbachol to terminal fields of the ascending mesolimbic cholinergic arousal system caused an increase in attention, vigilance, in the number of eye movements, dilation of pupils, increase in respiration rate and muscle tension, as studied in the cat [112], and caused a concurrent decrease in locomotor activity, increased immobility (freezing response), and induced a general behavioral inhibition both in cats and rats [113,114]. These symptoms were accompanied by sustained emission of aversive vocalizations, 22 kHz alarm calls in the rat [101,115] and threatening growling vocalizations in the cat [112]. The clusters of all the effects were interpreted as behavioral symptoms of anxiety, and emission of 22 kHz calls in rats as an evolutionary equivalent of crying in humans [116].

Intracerebral carbachol also increased activity of the sympathetic nervous system, increased the mean arterial blood pressure and heart rate [117,118,119], and caused an increase in glycogen synthesis and hyperglycemia [120,121]. Intracerebroventricular carbachol increased serum corticosterone levels in a dose-dependent way and increased hypothalamic noradrenaline levels [122,123]. Additionally, in general, carbachol elicited hormonal and metabolic responses like those to moderate stress, as studied in dogs [124]. Intrahypothalamic-preoptic injections of carbachol significantly increased core body temperature in normal ambient temperatures [125] but lowered hypothalamic temperature, suggesting a redistribution of blood [126]. The development of stress response with a set of physiological changes prepares the animal for response to approaching danger and potential fight with an opponent or response to other harm to the body. This preparatory behavior also includes a decrease in feeling pain (increase in pain threshold) as preparation for harmful danger.

Intrahypothalamic injection of carbachol in rats decreased formalin-induced pain in a dose-dependent manner [127]. Antinociception was also obtained from other brain sites, and an increased threshold for pain was obtained by stimulation of 119 sites of the midbrain and diencephalon with carbachol [128]. Antinociception was frequently associated with hyperexcitability to non-noxious stimuli [128]. Thus, the animal felt less pain but was more sensitive to any external stimulation. This response is expected at high emotional arousal state, particularly when the animal may expect violent struggle and fight. It was found recently that cholinergically induced antinociception may be further regulated by the orexinergic system [129].

The set of symptoms induced by cholinergic activation of the ascending mesolimbic cholinergic system for aversive emotional arousal allows for better understanding what emotional arousal entails. The widespread effects of this system are quickly changing the entire state of the organism from a usual functioning at rest to an alarm mode of varying intensity by activating the somatic, autonomic, and endocrine systems. The results presented in previous subsections indicate that this defensive alarm response is somehow associated with the drinking response. How drinking response, would contribute to this defensive behavior? 

## 7. Biological Role of Aversive Arousal-Induced Drinking Response

Thus far, carbachol-indued polydipsia did not serve the regulation of thirst but it could be a defensive measure induced by the activation of the ascending mesolimbic aversive arousal system. Rats overloaded their bodies with water regardless of homeostatic water balance needs. Moreover, drinking an additional load of water should increase diuresis as a mechanism for returning body water content to the original level. Instead, it was reported that injection of carbachol into the medial preoptic area induced antidiuresis, i.e., increased and retained water content in the body in a dose-dependent manner but controlling the electrolyte content [130]. A similar effect of antidiuresis induced by carbachol was recently confirmed and could be reversed by injection of catalase inhibitor into the rat septum [131]. These observations indicate that the biological role of the cholinergically activated drinking response is to increase water content in the body at least for the duration of the potential danger or duration of the aversive state. What benefits of higher water content in the body would be during an emotional defensive state?

There are several possible explanations to this question. Firstly, higher content of water in the organism would increase blood pressure [132,133]. This will increase blood perfusion in all organs, particularly in muscles and increase their oxygenation. Together with increased heart rate and glucose level in the blood (see Section 6 above), higher content of water would prepare the rat’s muscular system for increased effort. Another explanation might be that drinking additional fluids would increase overall blood volume as a defensive measure in anticipation of blood loss during intraspecies aggressive fight, although, fights among rats are highly ritualized and usually not associated with significant blood loss [134].

The excessive drinking response (polydipsia) might be only an occasional auxiliary behavior since rats might not have access to drinking water in face of a sudden danger in the natural environment. The most plausible alternative explanation is that the drinking response in rats caused by activation of the ascending aversive arousal system is an anxiety-induced response. It was suggested in earlier studies that the carbachol-induced responses represent anxiety type of aversive behavior, which is associated with stress response [116]. Thus, if water is available, drinking would represent anxiety-related response.

If this explanation is correct, then, more species would show anxiety/stress-induced drinking response. It was observed, however, that in many species studied for cholinergically induced drinking, the drinking response was not present (see Section 1). Anxiety- or stress-induced water drinking might not be a universal response, but some species should demonstrate it. Significant polydipsia was recently shown after chronic mild stress in mice without changes in sucrose intake [135]. Social stress in mice caused the development polydipsia or stress-indued overhydration with demonstrated trends of anxiety-like behavior [136,137]. Anxiety is a documented factor in this behavior both in animals and humans.

Polydipsia in humans is usually unrelated to the homeostatic regulation of water intake and is commonly observed in psychiatric patients [138,139]. A recent review has emphasized that polydipsic overhydration happens both in rodents and humans in response to anxiety and social isolation [140]. These results agree with the conclusion reached for the carbachol-induced drinking response in the rat. Long-lasting and severe polydipsia may, however, lead in humans to detrimental conditions termed “water intoxication” with secondary hyponatremia [140,141], so anxiety-induced polydipsia should be of short duration in physiological conditions. Thus, what benefit the organism has by a limited water overload in an anxiety situation?

It seems that the polydipsic behavior might be performed as a soothing procedure that decreases the aversiveness of the situation, so as a rewarding maneuver that contributes to alleviating the feeling of anxiety. Primary polydipsia was already suggested to occur as a stress-reducing behavior [142]. Counteracting the state of anxiety could happen by activation of the mesolimbic dopaminergic system originating from the ventral tegmental area and terminating in the nucleus accumbens. Activity of this rewarding system was documented to antagonize the cholinergically induced aversive state (anxiety) in rats [143]. Cholinergic projections can directly activate dopaminergic neurons.

The ascending cholinergic fibers were reported to synapse directly onto the dopaminergic neurons in the ventral tegmental area [144] and stimulation of the LDT neurons caused release of dopamine in the nucleus accumbens, a terminal field of the ascending dopaminergic projections [145,146]. These cholinergic connections to the dopamine neurons in the ventral tegmental area were suggested serving as a natural breaking mechanism during prolonged anxiety conditions. Studies of rat ultrasonic vocalization have shown that after induction of an intensive anxiety response by carbachol injected into the lateral septum with prolonged emission of 22 kHz vocalizations, a rebound effect has spontaneously occurred after several minutes (over 300 s). The rebound was characterized by sporadic emission of 50 kHz calls that were driven by the dopaminergic system, because they were antagonized by systemic haloperidol [147]. In other words, after several minutes of persistent cholinergically induced state, a rebound of dopaminergically driven episodes started gradually increasing up to the termination of the anxiety response. Cholinergic stimulation of dopaminergic VTA neurons is known to increase reward-seeking behavior and energize motor and locomotor behavior [148]. The cholinergically induced polydipsia could activate the dopaminergic neurons in the ventral tegmental area and bring some relieve of the carbachol-induced aversiveness and accelerate faster recovery from the induced anxiety.

## 8. Summary

Water drinking response in rats induced by direct cholinergic stimulation of vast subcortical limbic regions of the brain has been established in past studies. However, the biological and physiological roles of this well-documented pharmacological response have not been offered and convincingly interpreted for the last 50 years. The main difficulties in explaining the response were associated with observations that carbachol-induced drinking was not anatomically specific and could be induced from many subcortical limbic structures and could be antagonized by atropine from many sites other than injection sites. Moreover, the carbachol-induced drinking response was not comparable with physiological mechanisms of drinking, the renin-angiotensin system was not involved in this pharmacological response, and the response was not relevant to homeostatic water balance mechanisms. It is proposed that the cholinergically induced drinking response was initiated by activation of the ascending mesolimbic cholinergic system that has diffused nature and serves for induction of aversive arousal in the organism. Thus, the drinking response was associated with an anxiety-like state and had not served the regulation of thirst. This polydipsic behavior might be performed as a soothing procedure that decreases the aversiveness of the negative state induced by carbachol. It is, thus, concluded that water-drinking behavior is a rewarding procedure that contributes to alleviating the feeling of anxiety and bringing some relief from the carbachol-induced aversive state. Such polydipsia has been also documented in human patients. 

## Data Availability

Not applicable.
